# MetAnnotate: function-specific taxonomic profiling and comparison of metagenomes

**DOI:** 10.1186/s12915-015-0195-4

**Published:** 2015-11-05

**Authors:** Pavel Petrenko, Briallen Lobb, Daniel A. Kurtz, Josh D. Neufeld, Andrew C. Doxey

**Affiliations:** Department of Biology, University of Waterloo, 200 University Ave. West, Waterloo, ON N2L 3G1 Canada

**Keywords:** Metagenomics, Microbiome, Next-generation sequencing, Taxonomic profiling

## Abstract

**Background:**

Metagenomes provide access to the taxonomic composition and functional capabilities of microbial communities. Although metagenomic analysis methods exist for estimating overall community composition or metabolic potential, identifying specific taxa that encode specific functions or pathways of interest can be more challenging. Here we present MetAnnotate, which addresses the common question: “which organisms perform my function of interest within my metagenome(s) of interest?” MetAnnotate uses profile hidden Markov models to analyze shotgun metagenomes for genes and pathways of interest, classifies retrieved sequences either through a phylogenetic placement or best hit approach, and enables comparison of these profiles between metagenomes.

**Results:**

Based on a simulated metagenome dataset, the tool achieves high taxonomic classification accuracy for a broad range of genes, including both markers of community abundance and specific biological pathways. Lastly, we demonstrate MetAnnotate by analyzing for cobalamin (vitamin B_12_) synthesis genes across hundreds of aquatic metagenomes in a fraction of the time required by the commonly used Basic Local Alignment Search Tool top hit approach.

**Conclusions:**

MetAnnotate is multi-threaded and installable as a local web application or command-line tool on Linux systems. Metannotate is a useful framework for general and/or function-specific taxonomic profiling and comparison of metagenomes.

**Electronic supplementary material:**

The online version of this article (doi:10.1186/s12915-015-0195-4) contains supplementary material, which is available to authorized users.

## Background

Metagenomics has revolutionized the study of microbial communities, with major applications in numerous fields including microbial ecology, medicine, and biotechnology [1]. Whereas traditional approaches required culturing and microbiological characterization of individual microbial isolates, metagenomics involves sequencing and analysis of DNA fragments from the collective community of microbes present in an environmental sample. Metagenomic datasets capture both the taxonomic composition and the potential functional capabilities of microbial communities, exploring both “who is present?” and “what are they doing?”

As metagenomic datasets accumulate in size and sample throughput, bioinformatic analysis of the raw sequence data remains a considerable challenge. Major tasks include determining the taxonomic identity of sequenced fragments, the relative abundance of community members, the metabolic and physiological capabilities of these individuals, as well as the functions encoded by a microbial community in its entirety [2].

Two classes of methods exist for estimating microbial community abundance from metagenomic datasets: composition-based and identity-based methods. Composition-based methods, such as TETRA [3] and PhyloPythia [4], assign taxonomy to reads by comparing their composition (i.e., *k*-mer nucleotide profiles) to existing profiles from reference genomes. Composition-based methods have the advantage of being potentially applicable to sequences that lack homologs in reference databases but can be inaccurate when applied to shorter (<1,000 base) sequences [5].

Identity-based methods assign taxonomies through identification of similar sequences in reference databases. A standard approach is to search reference databases (e.g., National Center for Biotechnology Information’s (NCBI’s) RefSeq or non-redundant database) using Basic Local Alignment Search Tool (BLAST) [6], assigning taxonomy based on the best hit or lowest common ancestor of the most similar hits [7, 8]. Because the top BLAST hits are not always the nearest phylogenetic neighbors [9], these methods generally work well only when close homologs exist in databases [5, 7, 9]. Advanced methods therefore incorporate a phylogenetic approach into taxonomic classification [10–13]. The tools CARMA [10] and TreePhyler [12] scan metagenomic reads against the PFAM database [14] and build trees from the combined PFAM and metagenomic hits, thereby allowing the hits to be classified based on their phylogenetic placement relative to known reference annotations. The recently developed Phylosift [13] uses pplacer [15] to place identified metagenomic reads onto reference phylogenies pre-built with FastTree [16]. Additionally, hybrid methods for metagenomic taxonomic classification, such as PhymmBL [5], FCP [17], and others [18–20], combine both composition and reference sequence similarity, benefiting from the advantages of both approaches.

In addition to taxonomic profiling and estimation of community abundance from metagenomic data, a second class of methods exist for assessing the metabolic activities and pathways encoded by a microbial community. Commonly, functional annotation of individual or assembled reads can be performed by BLAST [6], and the collective set of functions are mapped onto reference pathway databases such as KEGG [21] or SEED [22] subsystems. More recent databases such as FunGene [23] and MetaPathways [24] have been developed with a focus on important “ecofunctional” gene markers and analysis pipelines relevant for environmental metagenomes.

Although existing tools are well equipped to assess the overall community composition or broad functional content of metagenomes, identifying the set of taxa that perform a particular *function* of interest within a metagenome remains a challenge. Moreover, often the biologist aims to analyze a particular gene or function that does not necessitate a large-scale, and often lengthy, analysis pipeline. This problem of *function-specific* taxonomic profiling is challenging because each function requires a custom analysis with a custom set of genes that may require a degree of user intervention and control. For a recent example, we assessed the microbial producers of cobalamin (vitamin B_12_) across a wide collection of 430 metagenomes from aquatic environments by targeting a customized collection of genes specific to the cobalamin synthesis pathway [25]. This process involved carefully selecting a set of genes/proteins representative of the pathway of interest, selecting a set of hidden Markov models (HMMs) representing those protein families, and searching for their homologs in a large number of metagenomes. We classified the hits taxonomically using methods described above and compared results among metagenome datasets. Owing to a lack of existing tools that automate this process and allow user flexibility and control, such analyses can be tedious and involve significant manual intervention.

Here we present MetAnnotate, a pipeline for function-specific taxonomic profiling and comparative analysis of metagenomes. MetAnnotate automates metagenome taxonomic profiling in the form of a user-friendly interface that can be installed either as a local, command line tool or as a web server for large-scale job handling. Using MetAnnotate, the user can choose any biological function, pathway, or set of proteins (represented as a set of HMMs), and these are scanned and taxonomically classified across selected metagenomes. MetAnnotate is therefore applicable to estimation of both function-specific and overall community relative abundance. MetAnnotate provides two separate taxonomic assignment methods: best hit assignment as well as phylogenetic placement onto reference trees that are uploaded or computed on the fly. The interface also facilitates easy comparison between metagenomes, thus highlighting functionally important changes in microbial community composition. To demonstrate the capabilities of MetAnnotate, we have benchmarked it on a commonly used simulated metagenome dataset, as well as used it to reproduce in a fully automated fashion the results of a previous analysis profiling aquatic cobalamin producers [25].

The project and open source code are available online at http://metannotate.uwaterloo.ca and https://bitbucket.org/doxeylab/metannotate, respectively.

## Implementation

### MetAnnotate pipeline and features

1. *Select query proteins/functions*

**Fig. 1:**
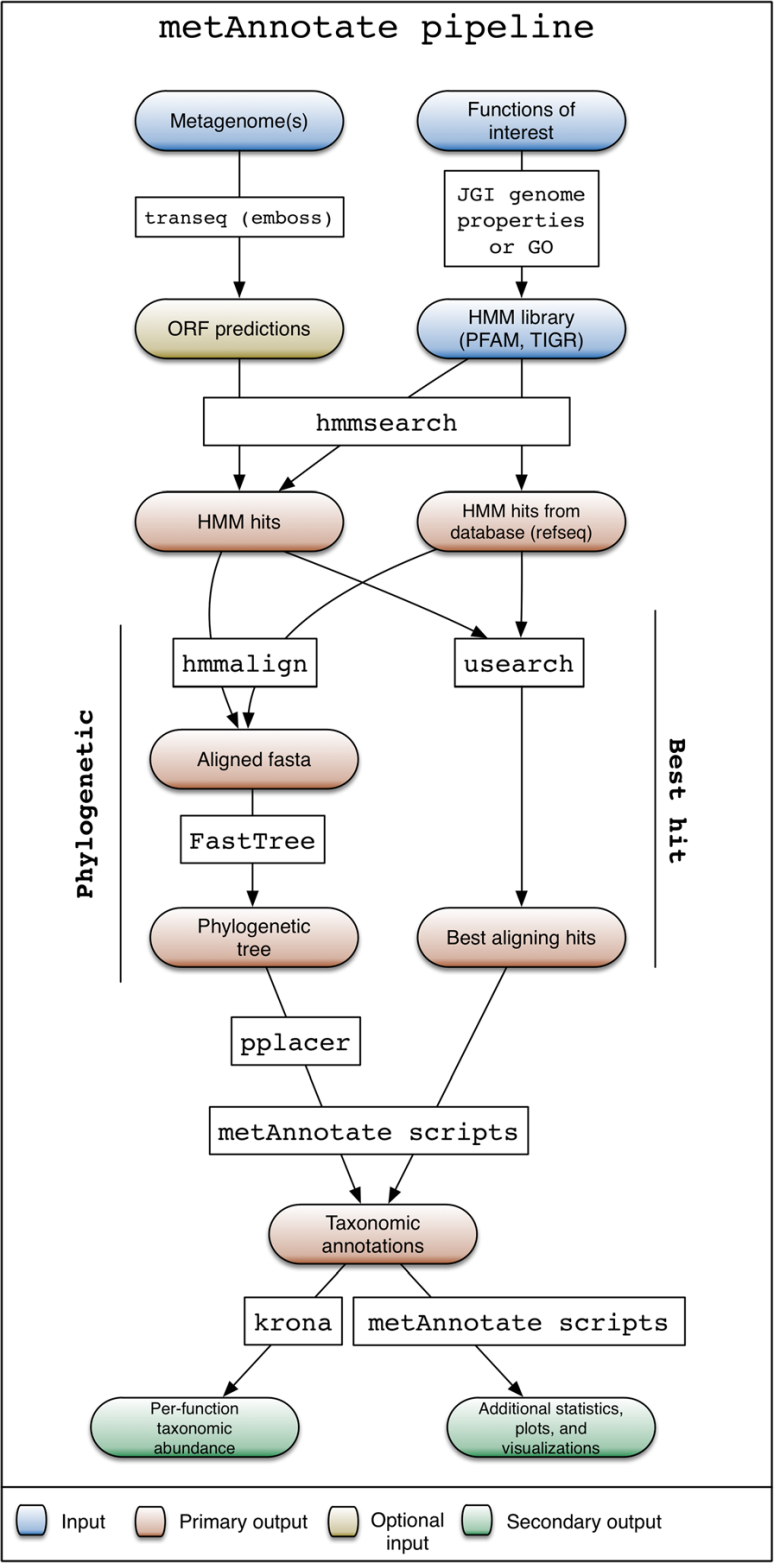
Backend MetAnnotate pipeline for Hidden Markov Model (*HMM*) search and taxonomic classification. *GO* Gene Ontology, *ORF* open reading frame

**Fig. 2:**
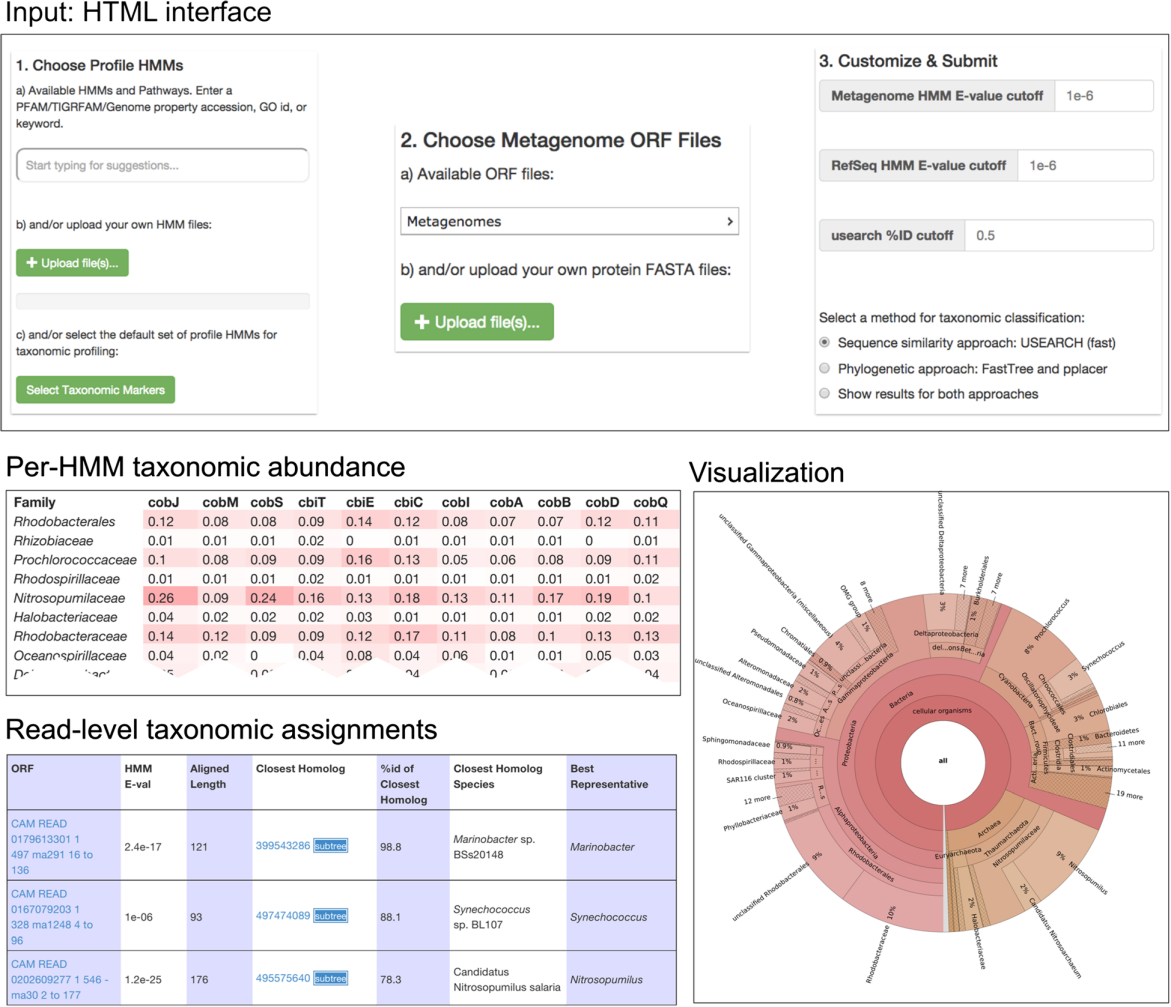
Screenshots of the MetAnnotate web interface

The HMM search and taxonomic classification pipeline (Fig. 1) begins with a user selecting from a set of available profile HMMs, or uploading HMMs of interest (Fig. 2, top). Available HMMs can be any PFAM [14] or TIGRFAM [26] protein families. Alternatively, a user can specify Genome Properties [26] or Gene Ontology (GO) identifiers [27] representing entire pathways or broader biological functions of interest, and the HMMs for different protein families attributed to that function are then retrieved automatically as queries. Functions and protein families are also searchable by keyword.

2. *Choose metagenomes for homology search*

The user then chooses metagenomes of interest. These can be in the form of unassembled metagenomic DNA sequences, protein sequences in a FASTA file, or a collection of pre-computed metaproteomes within a user-specified directory. If a nucleotide FASTA file is uploaded, it can be translated (6X) into open reading frames. Next, each HMM is searched via hmmsearch [28] against a reference database (NCBI RefSeq by default) to identify reference homologs, and against all user-selected metagenomes to identify metagenomic homologs. Hits are collected if *E*-values fall below a user-specified threshold (default *E* = 1e − 6).

3. *Taxonomic classification*

The metagenomic hits with detected similarity to the input HMM profile can then be taxonomically assigned using one or both of two approaches. In the default approach, the usearch [29] rapid sequence clustering algorithm is used to identify the best hit for each metagenomic homolog among the set of RefSeq homologs. Alternatively, phylogenetic classification can be done using an approach similar to that used by PhyloSift [13]. With this alternative approach, reference and metagenomic homologs identified by hmmsearch are re-aligned using hmmalign, and alignment positions corresponding to HMM match states are used to build a phylogenetic tree for reference homologs using FastTreeMP with default parameters. Trees are built on the fly but can be saved and uploaded as input for later runs. The identified metagenomic homologs are then placed onto the reference tree using pplacer, which is especially important because the aligned regions may differ. MetAnnotate reports the most common taxa at all taxonomic levels (e.g., genus, species) for the subtree containing the placed metagenomic sequence. Both pplacer (tree-based) and usearch (similarity search) approaches can be compared in the final result, giving the user flexibility regarding choice of taxonomic classification method.

4. *Results and visualization*

Once analyses have completed, the user may wish to obtain an overview of predicted taxonomic profiles, with the possibility of subdividing the results by metagenomic dataset and by query HMM. MetAnnotate provides three types of reports for this purpose (Fig. 2):

(I) Online (HTML) and offline (tab-separated) tables of detailed annotations for each metagenomic read
(II) Interactive Krona charts [30] summarizing taxonomic composition
(III) Heatmap tables of taxonomic abundance

(I) HTML table: this is the lowest level perspective, most appropriate for inspection of individual reads. The user can select the columns (i.e., annotations) they wish to view and sort by any feature. Individual reads can be viewed and their placed position within the phylogenetic tree can be displayed along with the reference hits used in classification of that read. Because different reads may be best assigned at different taxonomic levels, MetAnnotate can also estimate this level using the pplacer classification method by determining the lowest common ancestor in the read’s subtree that is present above a specified percentage (default is 80 %).

(II) Krona charts: these are most appropriate for a broad overview of the taxonomic composition of an individual metagenome dataset. These data displays (Fig. 2) allow for an interactive overview of the taxonomic profile as a “zoomable” pie chart. If multiple HMMs or datasets are used, Krona charts for each can be accessed quickly through a dropdown list for comparison.

(III) Heatmap tables: these are most appropriate for comparison of results between HMMs and datasets. The heatmap table (Fig. 2) shows the proportion of each taxa in each dataset, further subdivided by HMM. This facilitates a side-by-side comparison of taxonomic profiles for different metagenomes or HMM-specific functions. The user can choose the level of taxonomic analysis (e.g., class, genus, species) they wish to perform. These heatmaps are therefore useful for several applications: First, they may highlight differences in taxonomic composition between metagenomes. Second, they may reveal how different genes or functions are represented by different sets of taxa. Third, they may reveal how the taxonomic profile for a particular function may differ from overall community abundance.

Predicted annotations (tab-separated text files), trees (newick files), and multiple sequence alignments (aligned FASTA files) can be downloaded for further offline analysis.

## Results and Discussion

### Benchmarking and accuracy

**Fig. 3:**
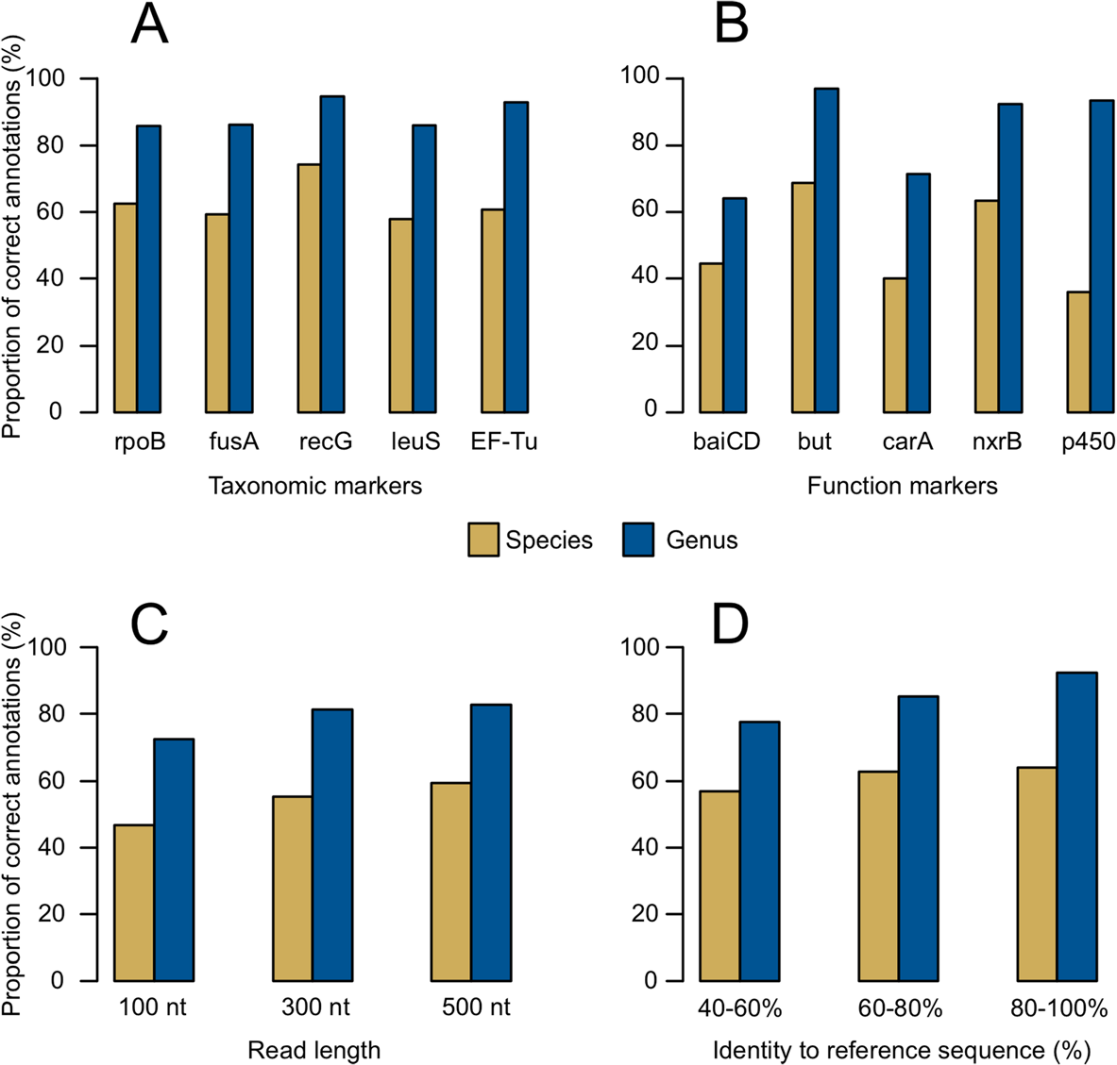
Taxonomic classification accuracy of MetAnnotate based on a simulated metagenome dataset and the best hit classification method. The proportion of correct taxonomic annotations assigned to detected homologs is shown for five different taxonomic markers (**a**) and five markers of biological functions (**b**), as well as different read lengths (**c**) and metagenomic-to-reference sequence identities (**d**). Results for (**c**) and (**d**) are based on all taxonomic marker homologs identified in (**a**)

To measure the accuracy of taxonomic predictions, we used MetAnnotate to analyze a commonly used benchmarking dataset, the Simulated High Complexity Metagenome (simHC; [31]). For query proteins, we selected a set of five taxonomic markers [32], as well as five markers of specific metabolic pathways chosen from the FunGene database [23]. We then measured the *precision* (i.e., fraction of reads annotated correctly) at multiple taxonomic levels (Table 1, Fig. 3). Unclassified reads were counted as incorrect predictions, but were rare occurrences (<5 %) and thus had a negligible effect on accuracy estimates.

**Table 1:** Taxonomic classification accuracy [proportion of correctly assigned sequences (%)] for MetAnnotate’s best hit and phylogenetic classification approach

Annotation method	Species	Genus	Phylum
*Taxonomic markers*			
Best hit	61.8	87.4	94.5
Phylogenetic	60.0	87.6	97.3
*Function markers*			
Best hit	47.4	78.7	83.3
Phylogenetic	46.2	80.8	90.1

For taxonomic markers, MetAnnotate assigned 61.8, 87.4, and 94.5 % of reads correctly at the species, genus, and phylum levels, respectively, using the usearch (best hit) method (Table 1, Fig. 3a). Using these markers, MetAnnotate also reproduced the known taxonomic composition of the simHC dataset with high accuracy (*r* = 0.82, Spearman correlation), which was greater than the accuracy obtained using four other methods [17, 20, 33], including the widely used MG-RAST [33] server (*r* = 0.75, Spearman correlation) (Additional file 1: Figure S1).

For markers of specific biological functions, which are likely to be more diverse and may exhibit uneven taxonomic distributions, MetAnnotate correctly assigned 47.4, 78.7, and 83.3 % of reads at the species, genus, and phylum level (Table 1, Fig. 3b). As expected, taxonomic classification accuracy also increased with read length (Fig. 3c) and sequence similarity to the best database hit (Fig. 3d).

#### Best hit versus phylogenetic classification

Because metagenomes containing a high proportion of novel sequences may be difficult to annotate using a best hit approach, MetAnnotate also includes an alternate phylogenetic classification method. Although slower and less accurate on short reads (Table 2), this method exhibits comparable accuracy to the best hit approach overall (Table 1), and is more accurate for novel sequences with lower sequence identity to the database (Table 2). Another major advantage is its ability to classify virtually all sequences (high sensitivity), whereas the usearch method will result in unclassified sequences when they are too dissimilar from the database (i.e., below 40–50 % identity). This can be a sizeable portion of a metagenome depending on its degree of taxonomic novelty [34].

**Table 2:** The effect of length and similarity to database on taxonomic classification accuracy (genus-level) using best hit and phylogenetic classification. Numbers indicate proportion of correctly assigned sequences (%)

	Best hit	Phylogenetic
*Length (nuceloteides)*		
100	72.3	58.1
300	81.3	81.2
500	82.7	81.3
*Sequence Identity to reference (%)*		
40–60	77.6	82.8
60–80	85.2	83.4
80–100	92.4	89.6

### Example application: fast taxonomic profiling of cobalamin producers in aquatic metagenomes

To demonstrate the capabilities of MetAnnotate, we replicated an earlier study assessing the taxonomic composition of aquatic cobalamin (vitamin B_12_) producers [25]. In our earlier study, 431 metagenome samples from a diverse range of aquatic habitats were scanned for 11 proteins in the cobalamin synthesis pathway. These hits were then annotated taxonomically by subsequent BLAST searches against the RefSeq database, a procedure that took several days of computing time on an eight-core Linux workstation.

Using an instance of MetAnnotate on the same resource, we reproduced the previously published analysis in under an hour (Fig. 2, bottom right). In addition, MetAnnotate reproduced previous results with a high degree of consistency. For instance, the four phyla highlighted previously as dominant cobalamin producers [25] were present in almost identical proportions in the current analysis: *Proteobacteria* (55 % previous, 55 % current), *Thaumarchaeota* (16 % previous, 15 % current), *Cyanobacteria* (14 % previous, 13 % current), and *Bacteroidetes/Chlorobi* (5 % previous, 5 % current).

Such substantial speed improvements stem from several heuristics used by MetAnnotate. First, all reference homologs of an HMM are identified initially in a single step, which avoids unnecessary re-computation. Second, pplacer and usearch annotation steps are significantly faster than BLASTp searches against the full database. Third, only reference HMM hits are used for database searching, which reduces database size.

#### Comparison between environments and between HMMs

**Fig. 4:**
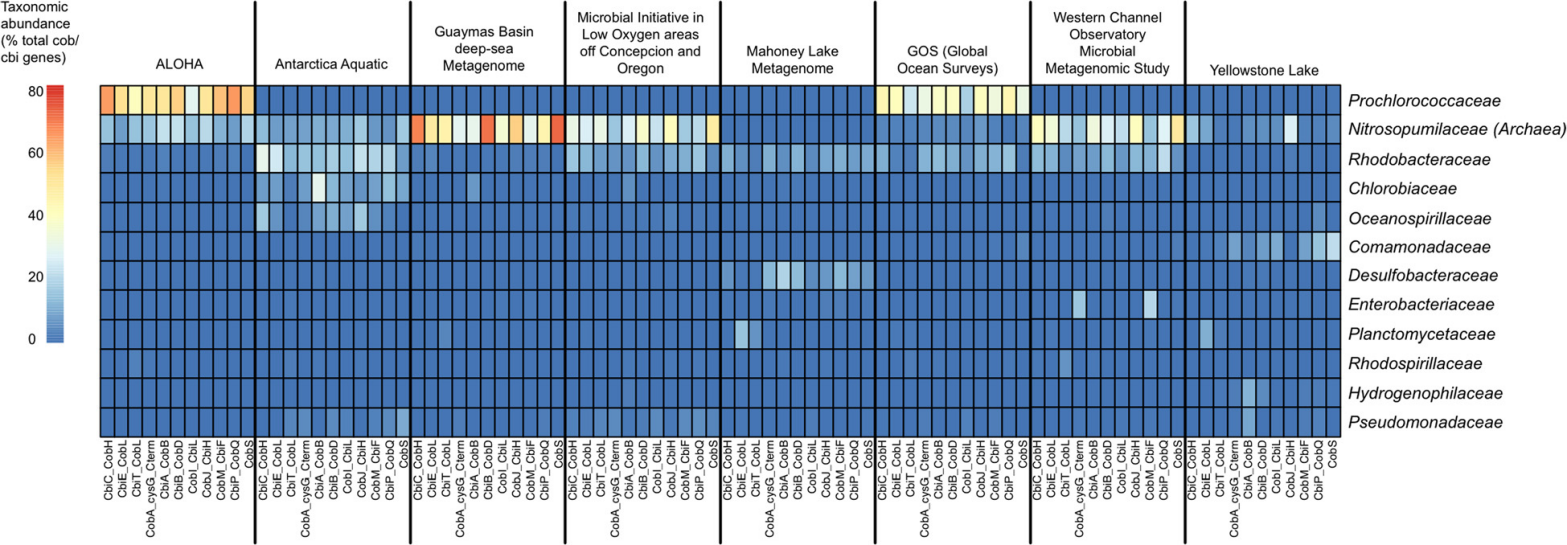
Example application: taxonomic profiling of cobalamin (vitamin B_12_) producers in aquatic metagenomes using MetAnnotate. Taxonomic profiles (family level) based on 11 cobalamin synthesis proteins are shown for eight metagenomes. See [25] for additional information

MetAnnotate predicted the taxonomic composition of cobalamin producers across eight metagenomes, and did so using 11 different genes within the cobalamin synthesis pathway (Fig. 4). Consistent with our previous results [25], this analysis revealed that the taxonomic composition of cobalamin producers was significantly different between sampled environments. For example, cobalamin gene representation was dominated by the family *Nitrosopumilaceae* (phylum *Thaumarchaeota*) in deep or polar environments, such as the Guaymas Basin Deep Sea Metagenome and the Microbial Initiative in Low Oxygen areas of Conception and Oregon (MILOCO) metagenome. In other sampled marine habitats, *Prochlorococcaceae* (phylum *Cyanobacteria*), *Rhodobacteraceae (*phylum *Proteobacteria*), or other taxa were the dominant sources of cobalamin genes.

In addition, this repeated MetAnnotate analysis demonstrated that the predicted taxonomic profiles were highly consistent between the 11 different cobalamin synthesis marker genes. This consistency provided independent verification of the predicted taxonomic profiles; the 11 separate profiles are averaged by MetAnnotate to provide a more accurate statistic of overall abundance. Offline, these data can be examined further to distinguish taxa contributing partial versus complete pathways.

### Novelty of MetAnnotate

Although there are numerous existing methods designed for metagenome community profiling [3–5, 10, 12, 13, 17–20, 33, 35], the novelty of MetAnnotate lies in its ability to perform custom analyses of taxonomic composition using any user-defined set of proteins. This makes it capable of both searching and taxonomically profiling specific biological functions across a large number of metagenomes. MetAnnotate has a range of novel features that distinguish it from other metagenome analysis tools: automated function selection (PFAM HMMs, GO terms and pathways), easy integration of custom HMMs, on-the-fly search and taxonomic classification, a built-in web server and queue capabilities for larger-scale job handling, and a web interface for comparative analysis and results visualization. A useful methodological feature is the ability to compare results from best hit and phylogenetic classification, each of which have their advantages. Overall, we anticipate that MetAnnotate will be useful in the functional and comparative analyses of shotgun metagenomes.

## Availability and Requirements

MetAnnotate is designed to run on Linux systems and is available at http://metannotate.uwaterloo.ca. Source code is available at https://bitbucket.org/doxeylab/metannotate, and an archived version is available at Zenodo [36]. Software is distributed under a MIT license. All computations reported in this manuscript were performed on a Lenovo Thinkstation E31 machine (Intel Xeon e3-1275v2 3.5 Ghz processor, 32Gb EC RAM) running Ubuntu Linux 14.04.1.

## Additional file

Additional file 1: Figure S1. MetAnnotate estimates microbial community abundance with high accuracy, demonstrated by estimated class-level taxonomic composition of the Simulated High Complexity Metagenome (simHC) dataset based on the taxonomic markers in Fig. 3a. The community abundance prediction made by MG-RAST (default parameters, LCA option) and three other methods are included for comparison. The Spearman correlations between known and estimated taxonomic abundance are: *r* = 0.82 (MetAnnotate); *r* = 0.75 (MG-RAST); *r* = 0.70 (MiniKraken); *r* = 0.65 (FCP NB-BL); *r =* 0.68 (FCP Epsilon-NB). (PDF 44 kb)

## Abbreviations

NCBI: National Center for Biotechnology Information
BLAST: Basic Local Alignment Search Tool
HMM: Hidden Markov Model
simHC: Simulated High Complexity Metagenome
GO: Gene Ontology
