# Supplementary figures and images for: MetAnnotate: function-specific taxonomic profiling and comparison of metagenomes
Source: BMC Biol. 2015 Nov 5;13:92. doi: 10.1186/s12915-015-0195-4 (PMC4636000; doi:10.1186/s12915-015-0195-4)

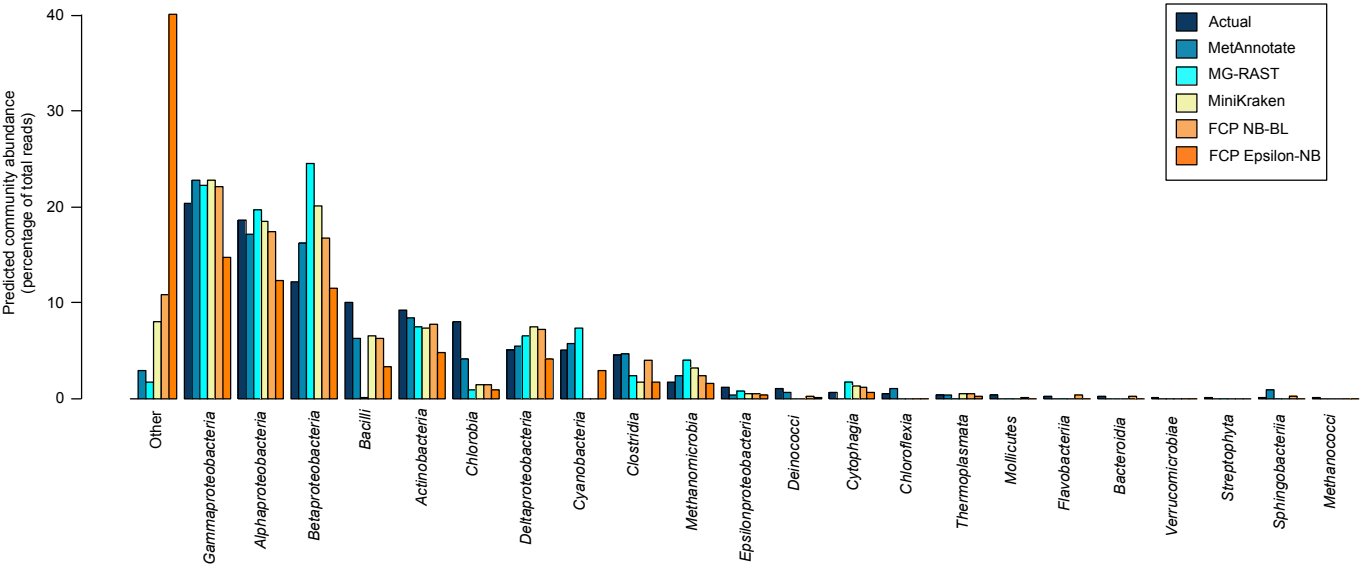

Supplement: Additional file 1: Figure S1. — MetAnnotate estimates microbial community abundance with high accuracy, demonstrated by estimated class-level taxonomic composition of the Simulated High Complexity Metagenome (simHC) dataset based on the taxonomic markers in Fig. 3a. The community abundance prediction made by MG-RAST (default parameters, LCA option) and three other methods are included for comparison. The Spearman correlations between known and estimated taxonomic abundance are: r = 0.82 (MetAnnotate); r = 0.75 (MG-RAST); r = 0.70 (MiniKraken); r = 0.65 (FCP NB-BL); r = 0.68 (FCP Epsilon-NB). (PDF 44 kb) [file 12915_2015_195_MOESM1_ESM.pdf]
